# *Dioscorea* spp. (A Wild Edible Tuber): A Study on Its Ethnopharmacological Potential and Traditional Use by the Local People of Similipal Biosphere Reserve, India

**DOI:** 10.3389/fphar.2017.00052

**Published:** 2017-02-14

**Authors:** Sanjeet Kumar, Gitishree Das, Han-Seung Shin, Jayanta Kumar Patra

**Affiliations:** ^1^School of Life Sciences, Ravenshaw UniversityCuttack, India; ^2^Research Institute of Biotechnology and Medical Converged Science, Dongguk University-SeoulGoyang-si, South Korea; ^3^Department of Food Science and Biotechnology, Dongguk University-SeoulGoyang-si, South Korea

**Keywords:** bioactive compounds, *Dioscorea*, ethnobotany, food values, ethnopharmacology, Similipal Biosphere Reserve

## Abstract

A number of wild crops remain unexplored in this world and among them some have excellent medicinal and nutritional properties. India is a harbor of biodiversity in general and phytodiversity in particular. The plant diversity is distributed from the Western Ghats to Eastern Ghats, along with the North-Eastern region and from the Greater Himalayas to the plain of Ganga. Among these distributed floral regions of the country, the Eastern Ghats are important due to their rich floral diversity. The forests of Odisha form a major part of Eastern Ghats in general and the Similipal Biosphere Reserve (SBR) in particular. The SBR is inhabited by many local communities. The food and medicinal habits of these communities are not fully explored even today. They are dependent on the forests of SBR for their food and medicine. Among their collections from forests, root and tuberous plants play a significant role. The local communities of SBR use about 89 types of tuberous plants for various purposes. *Dioscorea* is one such tuber, having maximum use among the local of SBR. However, less documentation and no specific reports are available on the food and medicinal values of the species available in this part of the World. *Dioscorea* species, popularly known as Yam worldwide and as Ban Aalu in Odisha, India, is a prime staple medicinal-food substitute for the majority of rural and local people of the state of India. Of the 13 *Dioscorea* species available in SBR, 10 species are known to be bitter in taste and unpalatable when taken raw. Since less documentation is available on the *Dioscorea* species of SBR and their traditional uses, the present study was focused on the ethnobotany, nutritional and pharmacological values of these species along its nutraceutical importance.

## Introduction

**Figure 1:**
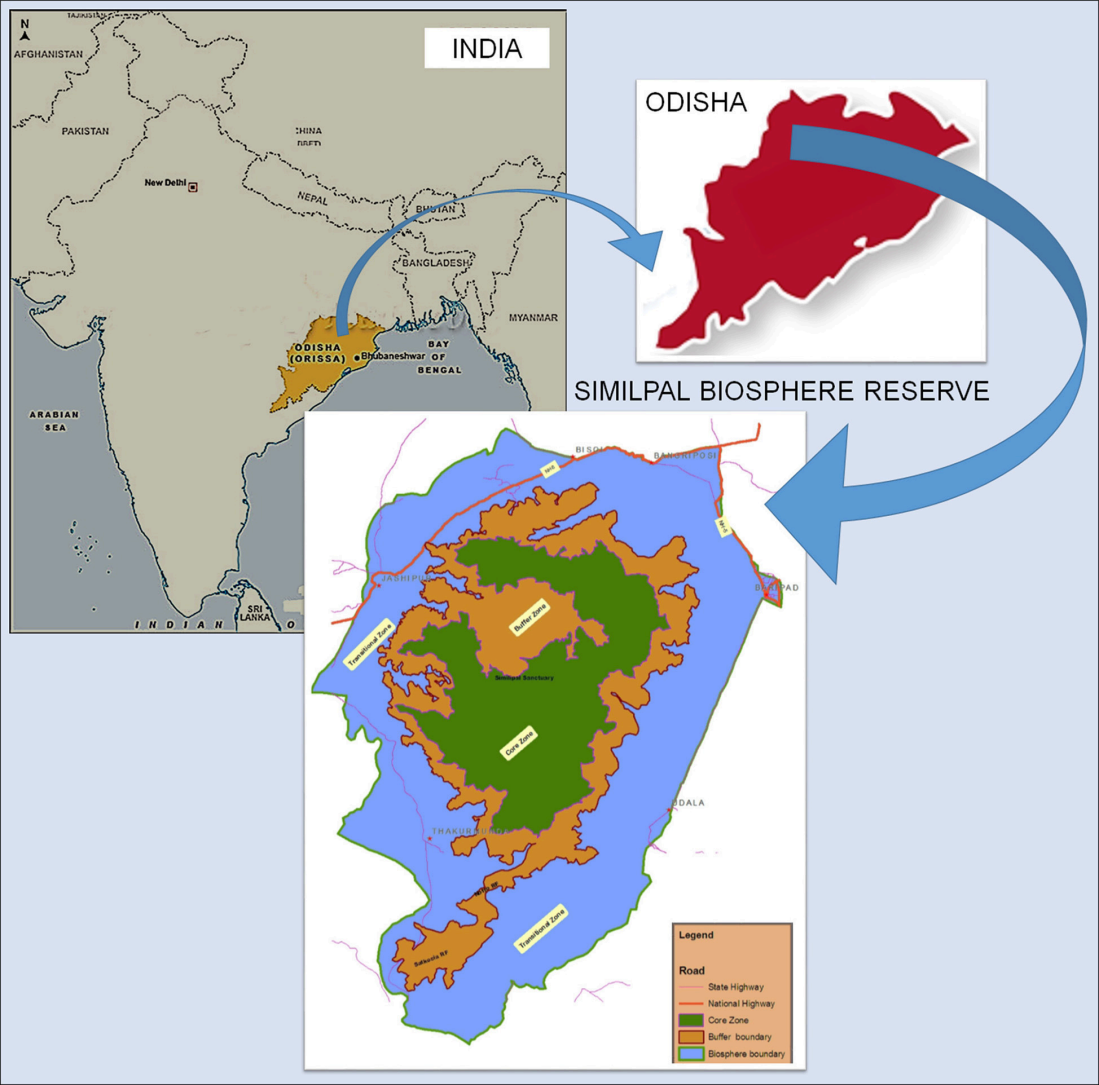
**Geographical location of the study area (Similipal Biosphere Reserve)**.

Food and health for all are major challenges for the developing countries this century. These challenges need to be addressed robustly. Scientific researchers can find practical solutions by searching for alternate sources of food and medicines. India is rich in phytodiversity, with about 45,000 plant species from the Western Ghats to Eastern Ghats along with the North-Eastern region and from the Greater Himalayas to the plain of Ganga. The forests of Odisha form a major part of the Eastern Ghats (Majumadar and Datta, 2015; Pellegrini et al., 2016). The state is also blessed with a biosphere reserve, Similipal Biosphere Reserve (SBR), which covers major part of Eastern Ghats (Figure 1). Besides having large floral biodiversity, SBR is inhabited by many local communities. The food and medicinal habits of these local people are unexplored even today. The forest resources available in this area are most often ill managed due to lack of awareness. In addition, there has been an increasing awareness about the nutritional status of the community, particularly regarding young children and nursing mothers, during recent years (Raju et al., 2015; Ahmed, 2016; Thongam et al., 2016). The Government of India and other organizations are carrying out a number of programs to create awareness; however, malnutrition remains challenge to rural and local communities of India. The condition is still worse in a state like Odisha in general and among the local communities of SBR in particular.

Appropriate nourishment needs to be pursued at household level, particularly in remote areas like SBR. The solution to these burning problems can be achieved through measured use of available natural resources. To fulfill their needs, people try to get their nutritional and medicinal requirements through nonconventional means, by consuming various wild plants and their parts (Singh and Arora, 1978). Such nonconventional sources are directly related to biodiversity. Nature provides numerous wild edible-medicinal products, such as leaves, flowers, fruits, nuts, berries, stems, roots, and tubers (Chandrasekara and Kumar, 2016; Geng et al., 2016; Hailu and Addis, 2016; Keservani et al., 2016). Among these, roots and tubers come under the minor wild crops, having very less scientific documentation, although they play important roles to fight against food scarcity. As wild tuber crops available in SBR, *Discorea* sp. (Ban aalu) species play a prime role in providing food and medicinal requirements for the local communities (Kumar et al., 2012, 2013a,b).

There are about 13 types of *Dioscorea* species found in SBR. The most common *Dioscorea* species are *D. bulbifera* L. (Pita aalu), *D. pentaphylla* L. (Panja Sanga), *D. hispida* Dennst. (Banya aalu), *D. alata* L. (Khamba aalu), *D. oppositifolia* L. (Paani aalu), *D. pubera* Blume (Kukai Sanga) etc. Ten species are known to be bitter in taste or unpalatable when taken raw. The rural and local people who use them as food supplements make them edible by different traditional practices. While investigating traditional food systems and palatability of *Dioscorea* species available in SBR, it was observed that tubers are mostly soaked overnight in water or left overnight in stream and subjected to successive boiling to remove the bitterness (Bhandari and Kawabata, 2005; Kumar et al., 2012; Misra et al., 2013). *Dioscorea* species with nutritive and antioxidant content not only enrich the diet of the local rural and local people but also make them ethnomedicinally important (Son et al., 2014; Chandrasekara and Kumar, 2016; Cui et al., 2016; Liu et al., 2016; Zhang et al., 2016). Tubers of different species of *Dioscorea* are used for curing various diseases and ailments in different formulations. Most of the tubers of *Dioscorea* examined in the present study are used for birth control and skin infections. Tubers and vegetative parts of these species are used either in single or in multiple formulations.

A harmonious blend of indigenous knowledge is therefore essential to document and promote proper utilization of such resources available in the state. In this context, the present study found some unique and unreported ethnobotanical claims of *Dioscorea* species available in SBR. Knowledge of local practitioners and millions of aboriginals from generation to generation flows into the mainstream, which has emerged as a traditional therapeutic system. However, the modern scientific mind cannot accept such local knowledge unless it is experimentally substantiated. The curative properties of medicinal and aromatic plants are due to the presence of certain bioactive compounds in them (Arunachalam et al., 2015; Atansov et al., 2015; Yamani et al., 2016). Through literature survey, the bioactive compounds present in the vegetative parts of some *Dioscorea* species were noted and concluded. The bioactive compounds presents in most of the *Dioscorea* species are in the phenolic group of compounds (Liu et al., 2011; Ghosh et al., 2012; Raju and Rao, 2012; Kumar et al., 2013a; Schulte et al., 2013; Saxena and Raja, 2014; Woo et al., 2014; Yang et al., 2014; Dzialo et al., 2016; Price et al., 2016; Van, 2016). Keeping all the properties of *Dioscorea* species available in SBR and utilized by the local community of SBR, an attempt has been made here to document the ethnobotanical and ethnopharmacological values of *Dioscorea* species used by the local communities of SBR, Odisha, India which would be helpful for researchers for utilization of this plant species in pharmaceutical applications and drug discovery.

## Similipal biosphere reserve

Similipal in Odisha state of India was designated as a biosphere reserve in June 1994, as the representative ecosystem under the Eastern Ghats. The SBR (Figure 1) has a unique assemblage of a number of ecosystems, such as mountains, forests, grasslands, and wetlands, which congregate into a contiguous patch with a range of diverse vegetation types. Its rich flora and fauna with many indicator species make the region a unique base for ecological studies. The biosphere reserve has varied topography and geologic formations, and rich biodiversity. It is also the habitat of many aboriginals (Das and Das, 2008).

The SBR is called the “Himalayas of Odisha” as it controls the climatic regime of parts of Odisha, Jharkhand, West Bengal, and Eastern India (Saxena and Brahmam, 1989). It harbors the largest tropical peninsular Sal zone forming a biological link between Northern and Southern India. The SBR is located in central part of Mayurbhanj district in Odisha state of India, close to the interstate boundary with West Bengal state in the North-East direction and Jharkhand state in the North-West (Misra, 1997; Girach et al., 1999; Behera, 2006; Reddy et al., 2007; Thatoi et al., 2008; Rout et al., 2009). It extends between 21°10′ to 22° 12′ N latitude and 85° 58′ to 86° 42′ E longitude, ranging between 300 and 1180 m above sea level. SBR is a firm mass of natural forest spread over a total area of 5569 km^2^ with core (845 km^2^) and buffer zones (2, 129 km^2^) composed of 16 forest ranges surrounded by a transitional zone (5, 569 Km^2^). The average elevation is 559.31 meters (Mohanta et al., 2006; Misra et al., 2011; Panda et al., 2011). The highest point in this group of hills is the Meghasani Hill (literally meaning *The Seat of Clouds*), which rises to about 1166 meters above sea level. Durdurchampa (1009 meters) and Chahala (775 meters) are the other important hills of the area (Saxena and Brahmam, 1989). Thus, SBR has idiosyncratic biodiversity harboring number of endemic, threatened, rare, medicinal, and economically important plants. Recent reports indicate, SBR has around 1254 species of vascular plants, representing 46% of the flora of Odisha and 7% that of India, including about 94 species of orchids, and about 52 species of rare/ endangered plants (Yoganarasimhan and Dutta, 1972; Swain and Nanda, 1997; Misra et al., 2003, 2011, 2013; Reddy et al., 2007; Rout and Thatoi, 2009; Mishra et al., 2011; Kumar et al., 2012).

## Local communities of similipal biosphere reserve and their traditional use of *Dioscorea* species

The extensive and densely forested hilly tracts of SBR are the home of many local communities, such as Bathudi, Bhumija, Gonda, Ho, Kolha, Mahali, Munda, Pauri Bhuiyan, Santhal, and Saunti, including two primitive groups, Hill-Kharia and Mankirdia (Saxena and Brahmam, 1989; Pandey et al., 2000; Pandey and Rout, 2003; Mohanta et al., 2006; Mishra, 2010; Kumar et al., 2012; Misra et al., 2013; Pedi et al., 2013; Sahoo, 2013). There are around 61 villages inside the core and buffer zone and about 1200 villages in the transitional zone, having a total population of about 4,50,000, out of which the scheduled tribes constitute 73.44 % of the total population of Similipal (Mishra, 2010; Mishra et al., 2011; Upadhyay et al., 2012). These poor local peoples practice primitive culture, traditions, and rituals, and have no or very few acquired skills. Their main occupation is food gathering, hunting, collection of forest products, and traditional farming or agriculture. In the present study, the Ho, Bathudi, Mankirdia, Kolho, Munda, and Santhal were selected for detailed study on their traditional knowledge on *Dioscorea* species.

The “Ho” is a Kolarian ethnic group belonging to the same stock of Munda and Kol. They mainly cultivate rice, maize, and millets along with seasonal vegetables. They also collect different types of wild plants from the forest and store them (Ota et al., 2013). They usually collect tubers and rhizome, including starchy tubers of *Dioscorea* species. They use tubers as food and medicine (Tables 1, 2). They are inhibiting from collecting *D. pubera* Blume (Figures 2.3, 2.5) during rainy seasons. Mankirdia is a primitive tribe that constitutes a semi-nomadic section of the Birhor tribe (Ota, 2007; Dash, 2011). They are primarily a hunting and food gathering community (Ota and Mohanty, 2008). The present study observed that they wander from Similipal to Hazaribagh National Park (Jharkhand) and return after a year to Similipal again (Source: Token Mankirdia, interviewed near the Kalikaparsad gate, transitional zone of SBR). During their movement in forest, they collect various types of medicinal plants to cure common diseases. They collect tuber of *D. bulbifera* L. (Figure 2.2) and *D. pentaphylla* L. (Figure 2.1) for curing skin infections, and abdominal pain, and for birth control (Kumar et al., 2012; Misra et al., 2013). They also collect tubers during early winter and store them for consuming in the summer and rainy seasons. The Hill-Kharia, locally known as “Pahari Kharia” is a highland local group (Ota et al., 2008). They are expert in collection of honey, resin, and arrowroot. They are primarily a forager community in the SBR. They do major seasonal collection along with agricultural labor in the agricultural season (Ota and Sahoo, 2013). During the rainy season, most of the Hill-Kharia face rice scarcity, and they principally depend on other food stuffs, like maize, edible roots, and tubers of *Dioscorea* species and corns. The Santhals, one of the common local communities of India, mainly inhabit in the districts of Mayurbhanj, Keonjhar, and Balasore in the state of Odisha, India. They collect minor forest products like tubers, roots, fruits, green leaves, honey, mahua flowers, etc., that sustain them for 3–4 months in a year (Ota and Patnaik, 2014). Bathudi is also a very common local community in SBR. They are simple and shy in nature. They are excellent at agriculture and gathering forest products and medicinal plants. A detailed study on *Dioscorea* species is presented below.

**Table 1:** **Ethnobotanical values of common ***Dioscorea*** species of SBR, India**.

**Botanical name**	**Plant parts**	**Ethno-botanical/Pharmacological values**	**Supporting literature**
*Dioscorea alata* L.	Tuber	Tuber powder is used to cure piles.	Jadhav et al., 2011
	Tuber	Tubers are eaten raw twice a day until weakness is reduced.	Kamble et al., 2010
	Tuber	Juice of tuber is used to kill stomach worm.	Samanta and Biswas, 2009
*Dioscorea belophylla (Prain) Voigt ex Haines*	Tuber	Tuber juice with hot water is given to treat fever, malaria, headache, and dysentery.	Srivastava and Nyishi, 2010
*Dioscorea bulbifera* L.	Tuber	Raw tuber is eaten to enhance appetite.	Mishra R. K. et al., 2008
	Tuber	Tuber is good for intestinal colic, relieving dysmenorrhoea, reducing acidity, against rheumatoid arthritis, to relieve intense inflammation in the acute phase, in spasmodic asthma, for menopausal problems, for labor pain and the prevention of early miscarriage, for hernia, relieving the pain of childbirth.	Nayak et al., 2004; Patil and Patil, 2005; Bhogaonkar and Kadam, 2006; Mehta and Bhatt, 2007
	Leaves	Paste is used against skin infections.	Girach et al., 1999
	Tuber	Bulbils are used to reduce throat pain.	Mbiantcha et al., 2011
	Tuber	Boiled tubers are taken orally to reduce body heat.	Singh et al., 2009
	Tuber	Used against boils and dysentery.	Nag, 1999
	Tuber	Tuber powder mix with butter is given to check diarrhea.	Jadhav et al., 2011
	Tuber	Used as refrigerant to reduce body heat during summer.	Dutta, 2015
	Tuber	Used to treat skin infection.	Tiwari and Pande, 2006
	Tuber	Used to treat bronchial cough and used as antiseptic.	Bhatt and Negi, 2006
	Tuber	Useful for acidity and ulcers.	Dutta, 2015
	Stem	Tender shoots and twigs are crushed and rubbed on wet hair to remove dandruff.	Dutta, 2015
	Tuber	Root paste mixed with cow milk is taken orally for the treatment of cough and asthma.	Teron, 2011
	Tuber	Used to treat typhoid with *Curcuma aromatica*	Jain et al., 2008
	Tuber	Tubers are used in ulcer, piles, syphilis, and dysentery, and powder used to kill hair lice.	Abhyankar and Upadhyay, 2011
	Tuber	About 10 gm of powder is given once a day for 5–6 days after menses as contraceptive.	Kamble et al., 2010
	Tuber	Tubers are boiled after processing and given for abdominal pains. The tubers are dried and pea sized pieces are cut and given in early morning with water for 3 days to cure piles.	Abhyankar and Upadhyay, 2011
	Tuber	The roasted and mashed tubers are eaten with salt to cure cough.	Singh et al., 2009
*Dioscorea dumetorum* (Kunth) Pax	Tuber	Tuber juice is used to make arrow poison.	Edison et al., 2006
	Tuber	Used against jaundice.	Edison et al., 2006
*Dioscorea esculenta* (Lour.) Burkill	Tuber	Tubers are used for treatment of chest pain, nervous disorders, and swellings.	Edison et al., 2006
	Tuber	Tuber paste is used to relieve pain and to treat boils, dysentery and swellings.	Dutta, 2015
*Dioscorea hamiltonii* Hook.f.	Tuber	For treatment of stomach ache.	Edison et al., 2006
	Tuber	Eaten for poor appetite.	Edison et al., 2006
	Tuber	Crushed tubers are given as body refrigerant during summer seasons and good for treating diarrhea.	Sharma and Bastakoti, 2009
*Dioscorea hirtiflora* Benth.	Tuber	Used to treat gonorrhea.	Sonibare and Abegunde, 2012
*Dioscorea hispida* Dennst.	Tuber	Water of soaked tuber is used as medicine for eyes.	Meena and Yadav, 2011
	Tuber	Used as fish poison.	Nashriyah et al., 2011
	Tuber	Sap of tuber is pasted around the affected parts and covered with cloths for about one night to treat peeling of skin of feet.	Sharma and Bastakoti, 2009
	Tuber	Tuber is used to treat vomiting, indigestion, possesses narcotic properties and fresh tuber taken as purgative.	Dutta, 2015
	Tuber	Tubers are roasted and pounded and its paste is applied on wounds and injuries.	Kamble et al., 2010
*Dioscorea kamoonensis* Kunth	Tuber	Tubers are used in the treatment of arthritis and rheumatism.	Edison et al., 2006
*Dioscorea oppositifolia* L.	Tuber	Tuber is boiled with *D. uniflorus* and is given to women once a day for nearly a month after delivery to revive their strength.	Mishra S. et al., 2008
	Tuber	Oral administration of tuber powder mixed with honey is used for increasing sperm.	Sharma and Bastakoti, 2009
	Leaf	Leaf paste is used as antiseptic for ulcers.	Sheikh et al., 2013
	Tuber	Powered root mixed with cow urine is applied on scorpion bite.	Nashriyah et al., 2011
	Tuber	Leaves are mixed with leaves of clematis and 2–3 drops of juice put in the nose of affected person to get relief after sneezing in fits and epilepsy.	Kamble et al., 2010
*Dioscorea pentaphylla* L.	Tuber	Tubers are applied on swelling of joints and used as tonic to improve body immunity.	Edison et al., 2006
	Tuber	Used for stomach pain.	Choudhary et al., 2008
	Tuber	Crushed mass of tuber is given to cattle when they become sick by eating green leaves of maize.	Sharma and Bastakoti, 2009
	Tuber	Tuber is used as tonic and also used to cure stomach troubles and rheumatic swellings.	Dutta, 2015
	Tuber	Inflorescence is used as vegetables for body weakness.	Kamble et al., 2010
	Tuber	Tubers are useful to allay pain and swelling.	
*Dioscorea pubera* Blume	Bulbils	Bulbils are cooked and taken to cure colic pain.	Sheikh et al., 2013
*Dioscorea wallichii* Hook.f.	Tuber	Roasted and eaten for flatulence.	Dutta, 2015
	Tuber	Used in stomach pain.	Rout and Panda, 2010
*Dioscorea spinosa* Burm.	Tuber	Tubers are edible in the district Mayurbhanj of Odisha, India.	Behera, 2006

**Table 2:** **Common medicinal uses of ***Dioscorea*** species available in SBR, India**.

**Botanical name**	**Plant parts**	**Common medicinal properties**	**Supporting literature**
*Dioscorea bulbifera* L.	Tuber	Piles, dysentery, syphilis, and ulcers	Dutta, 2015
	Tuber	Contraceptive	Swarnkar and Katewa, 2008
	Tuber	Cough, leprosy, and diabetic	Dutta, 2015
	Tuber	Rheumatism	Sahu et al., 2010
	Tuber	Tuberculosis	Sharma and Bastakoti, 2009
	Tuber	Birth control	Coursey, 1967
	Tuber	Leprosy	Sharma and Bastakoti, 2009
	Tuber	Pig cysticerosis	Sharma and Bastakoti, 2009
	Tuber	Sore throat and struma	Sharma and Bastakoti, 2009
	Bulbils	Cuts, sores, abscesses, boils, and wound infection	Sharma and Bastakoti, 2009
	Bulbils	Piles, dysentery, syphilis, ulcers, pain, and inflammation	Sharma and Bastakoti, 2009
	Tuber	Piles	Behera, 2006
	Tuber	Muscular pain	Sahu et al., 2010
	Tuber	Hernia and scorpion sting	Dutta, 2015
	Tuber	Poor appetite	Mishra S. et al., 2008
	Tuber	Sexual vigor food	Radha et al., 2013
	Tuber	Wormicide	Das et al., 2003
	Tuber	Bone fracture	Padal et al., 2012
	Tuber	Bone fracture	Padal et al., 2012
	Tuber	Asthma	Choudhary et al., 2008
	Tuber	Infected skin	Kumar et al., 2013b
*Dioscorea hamiltonii* Hook.f.	Tuber	Piles	Mishra S. et al., 2008
*Dioscorea hispida* Dennst.	Tuber	Antidote against arrow poison	Edison et al., 2006
	Tuber	Peeling of skin of feet	Sharma and Bastakoti, 2009
	Tuber	Blood glucose	Harijono et al., 2013
	Tendrils	De-worming	Harijono et al., 2013
*Dioscorea oppositifolia* L.	Tuber	Swelling, scorpion stings, and snake bites	Edison et al., 2006
	Tuber	Oral administration of tuber powder mixed with honey for increasing sperm.	Neelima et al., 2011
	Tuber	Increase sperm number	Radha et al., 2013
	Tuber	Fracture	Padal et al., 2012
*Dioscorea pentaphylla* L.	Tuber	Abdominal pain after delivery	Swarnkar and Katewa, 2008
	Tuber	Diphtheria in cattle	Sharma and Bastakoti, 2009
	Tuber	Fracture	Padal et al., 2012
	Tuber	Stomach pain	Choudhary et al., 2008
	Tuber	Digestive tract problems	Choudhary et al., 2008
	Tuber	Skin infections	Dutta, 2015
*Dioscorea pubera* Blume	Tuber	Weakness	Kumar and Satpathy, 2011

**Figure 2:**
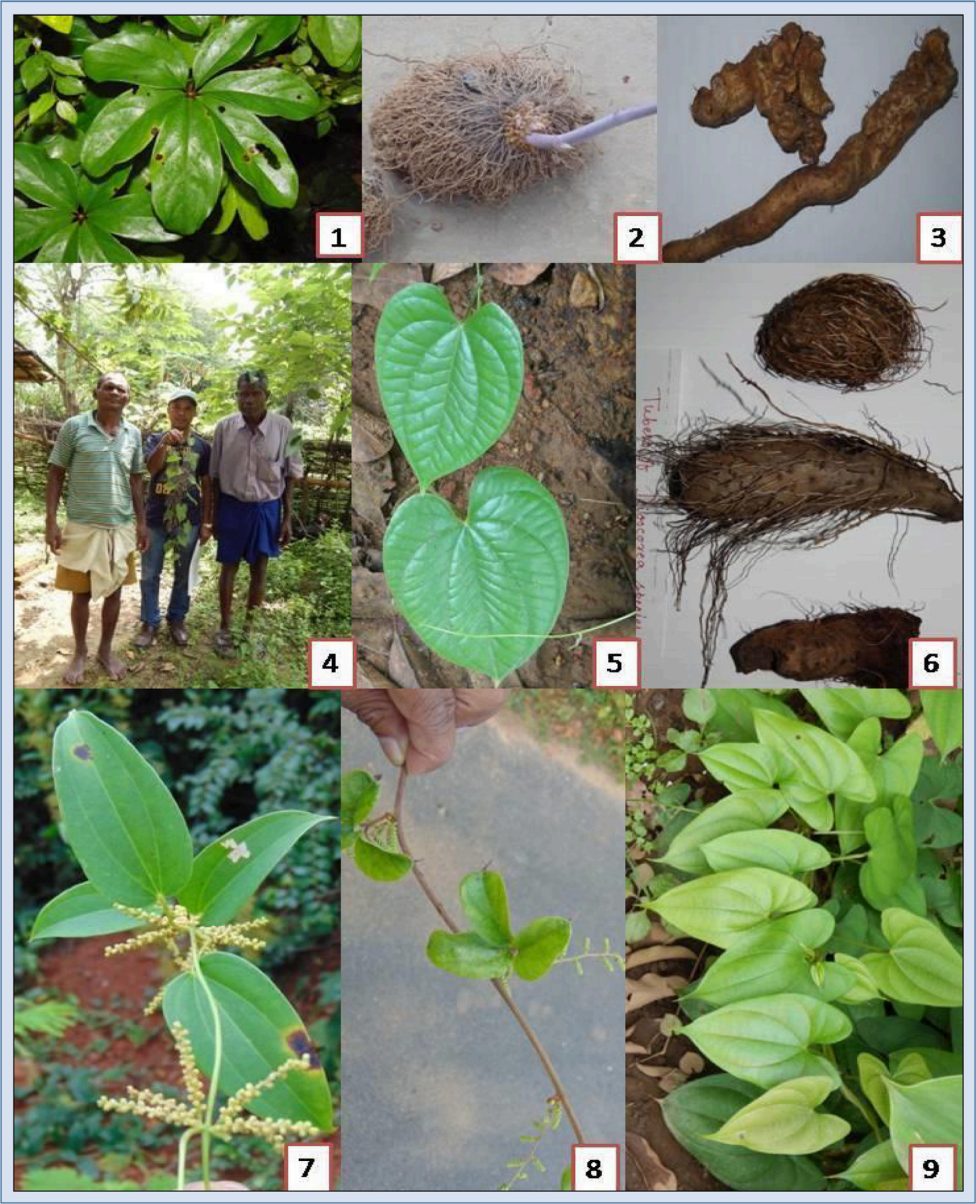
**Some common ***Dioscorea*** species of Similipal Biosphere Reserve. (1)** *D. pentaphylla* leaves, **(2)** Tuber of *D. bulbifera*, **(3)** Tubers of *D. puber*, **(4)** *D. bulbifera* with tribal communities of SBR, **(5)** *D. puber* leaves, **(6)** Tuber of *different Dioscorea* spp., **(7)** Flowers of *D. oppositifolia*, **(8)** Flowers of *D. hispida*, and **(9)** *D. alata*

## *Dioscorea* species: the wild tuber crop

### Origin, habitat, and distribution

The genus *Dioscorea* belongs to the family *Dioscoreaceae*, the most prominent within the order *Dioscoreales* (Burkill, 1960; Ayensu and Coursey, 1972; Dansi et al., 1999; Tamiru et al., 2008; APG III, 2009). The family is believed to be one among the earliest angiosperms, and it probably originated in Southeast Asia (Coursey, 1967). The various *Dioscorea* species apparently followed a divergent evolution in three continents separated by the formation of the Atlantic Ocean and desiccation of the Middle East (Hahn, 1995). As a result, this major species occur in three isolated centers: West Africa, Southeast Asia, and Tropical America (Alexander and Coursey, 1969). These centers are also considered areas for independent yam domestication, and represent considerable diversity (Asiedu et al., 1997).

Among the selected *Dioscorea* species, the most economically important species, *D. alata* L., originated in Southeast Asia—more specifically, in Tropical Myanmar and Thailand (Orkwor et al., 1998; Tamiru, 2006), and currently it is the most diversified and extensively distributed species. It is distributed throughout the Tropics, Southeast Asia, Papua New Guinea, Indonesia, Malaysia, Philippines, South Pacific Islands, Vanuatu, West Africa, Brazil, the Caribbean, South America, Central America, Florida, Escambia, Gadsden, Leon, Levy, and Charlotte (Gooding, 1960; Coursey, 1967; Lebot et al., 1998, 2006; Mignouna and Dansi, 2003; Scarcelli et al., 2006; Ahmad and Uroj, 2008; Hgaza et al., 2011). The other most popular among studied *Dioscorea* species is *D. bulbifera* L. The native range of this species is vast, and includes much of Asia, tropical Africa, and Northern Australia (Coursey, 1967; Terauchi et al., 1991; Onwueme and Charles, 1994).

Another species, *D. bulbifera* L. (Figure 2.4) is native to South Asia and distributed in Central and South America, Africa, Australia, Louisiana, Texas, Hawaii, Puerto Rico, Polynesia, Florida, and West Africa. (Coursey, 1967; Schultz, 1993; Langland and Burks, 1998) while *D. hispida* Dennst. (Figure 2.8) is distributed in Tropical and Sub-tropical regions, Philippines, China, Taiwan, Malaya, New Guinea, Malaysia, Fujian, Bhutan, Indonesia, Bangladesh, Sikkim, Thailand, Cambodia, Laos, Myanmar, Vietnam, and Africa (Sharma and Bastakoti, 2009; Nashriyah et al., 2011). *D. pubera* Blume is native to the Indo-China region (Asiedu et al., 1997) and *D. pubera* Blume is distributed throughout the temperate, tropical Americas, China, wet regions of Himalayas, Central Nepal, Western Malaysia, and Bhutan (Coursey, 1967).

The other most important highly yielding (tuber size) species is *D. pentaphylla* L. and it is native to Tropical Asia and Eastern Polynesia and is distributed in South-Eastern Asia, Tropical Asia, Kauai, Molokai, Hawaii, Tahati, Savii, North America, and Florida (Ayensu and Coursey, 1972). These species are distributed in most parts of the world and throughout the country of India. In India, the populations of these species are rich in Eastern and Western Ghats. The distribution of *Dioscorea* species selected for the present study is given below.

In India, the most common *Dioscorea* species is *D. alata* L. (Khambha Aalu) distributed throughout the country, and rich in Odisha. In SBR the species is dominating in peripheral areas such as Jashipur, Karanjia, Hatibadi, Manda, and Bisoi (Kumar et al., 2012) and *D. pubera* Blume (Kukai Sanga) is distributed mainly in areas like the Himalayas, Sikkim, Assam, Northern Bengal, Western Ghats, Jharkhand, Bihar, Upper Gangetic Plain, Kerela, Wayanad, Udupi, and Andhra Pradesh of the country. In SBR, its population is rich in Kasipani, Kolha, Gurguria, and Ghatkumari. It is very frequent in Bangriposi Ghati and Gurguria forest range (Saxena and Brahmam, 1995; Kumar et al., 2012; Misra et al., 2013).

The second richest *Dioscorea* species after *D. alata* L. is the *D. bulbifera* L. (Pita Aalu). It is distributed mainly in the forest patches of Eastern Ghats to the lower Himalayas in the Indian subcontinent. In SBR, It is widely distributed in Bangriposi Ghati, Gurguria, Sanuski, Nawana, Bakua, Kukurbhuka, Kalika Parsad and surrounding areas (Kumar et al., 2012) while *D. pentaphylla* L. (Panja Sanga) is distributed in Deccan, Western Himalayas, Rajasthan, Nasik, and Odisha states of the country. In SBR, it is rich in the hill slopes of Gurguria, Sanuski, Bangriposi, Ghatkumari, Astakumar, Nawana, and Joranda (Kumar et al., 2012). *D. hispida* Dennst. (Banya Aalu) is distributed throughout the mountainous areas of the country. It is very common in Odisha, Jharkhand, Andhra Pradesh and Telangana states. In SBR, it is distributed in the shady buffer and peripheral areas and along the streams, particularly in Ghatkumari and Padampur (Kumar et al., 2012). The above all information show the rich distribution of these species not only in the India but throughout the world.

## Botany of *Dioscorea* sp.

The word “Yam” is applied to members of the genus *Dioscorea* belonging to the family *Dioscoreaceae* in the order *Dioscoreales* (Alexander and Coursey, 1969). Wild species are either annuals or semi-perennials or perennials. Cultivated species are annuals. Generally, the female plants are less in number than the male plants. Most *Dioscorea* species have simple, cordate, or acuminate leaves borne on long petiole, but in some species, they are lobed or palmate with pointed tips. They are climbers and climb by twining. The direction of twining of the vine (i.e., anti-clock wise or clock wise) is a characteristic peculiar to the particular sections within the genus *Dioscorea*. The wings present in some species, such as *D. alata* L. (Figure 2.9), support the twining habit. The flowers are basically dioecious, with male and female flowers borne separately or on separate plants. The male or female flowers are borne on axillary spikes in the leaf axils. The male flowers are sessile, glabrous, and spherical and are borne axially or terminally. These flowers consist of calyx of three sepals and corolla of three petals, arranged regularly and almost similar in size and appearance, with three or six stamens (Onwueme, 1978). Fruits are mostly capsules. The seed in each capsule is small and has wings that vary in shape in different species (Onwueme, 1978). The seeds are flat and light, and the wings help in wind dispersion. Some species, such as *D. alata* L., *D. bulbifera* L., *D. pentaphylla* L., *D. pubera* Blume, have bulbils in the axils. Bulbils are specifically adapted for vegetative propagation (Coursey, 1967). They are very smaller than the underground tubers. Short day length generally accelerates formation of bulbils. *Dioscorea* possess shallow fibrous root systems, normally un-branched and concentrated within the top layer of the soil, and very few actually penetrate up to 1 m depth (Onwueme, 1978). The tuber is the storage organ, which forms a new tuber and shrivels away simultaneously when the re-growth is induced. When the organ lacks the typical characteristics of a modified stem structure, the tuber has no preformed buds or terminal bud at the distal end (Hahn et al., 1987).

## Ethnobotanical values of *Dioscorea* sp.

*Dioscorea* have sound ethnobotanical values throughout the Tropics. There are numerous reports available on local claims on *Dioscorea* species worldwide. In some forest areas of Southern Thailand, which are situated in the Tropical rain forest zone of Southeast Asia, local people use *Dioscorea* species to treat warts (Maneenoon et al., 2008). The boiled tubers of *D. membranacea* Pierre ex Prain & Burkill are used to treat asthma and fever (Maneenoon et al., 2008). The mucilage from the tubers of *D. piscatorum* Prain & Burkill is used to poison fish is used by the native people of Malaysia as a piscicide (Burkill, 1951, 1960). *D. prazeri* Prain & Burkill is used as soap and shampoo to kill lice in India (Maneenoon et al., 2008). *Dioscorea* is used in curing gastritis among Yoruba local groups of Cuba (Kadiri et al., 2014). Tubers of *D. hamiltonii* Hook.f. are used as body refrigerant during summer and are also used to treat diarrhea (Dutta, 2015). *D. bulbifera* L. is used against tuberculosis and raw tuber of *D. pentaphylla* L. against diphtheria in cattle (Sharma and Bastakoti, 2009). Tubers of *D. oppositifolia* L. (Figure 2.7) are used in the treatment of swellings, scorpion stings, and snake bites (Dutta, 2015). Juice of *D. wallichii* Hook.f. is used in the treatment of Jaundice. *D. hispida* Dennst. (Figure 2.6) is used as an antidote to arrow poison (Sinha and Lakra, 2005; Edison et al., 2006; Mishra S. et al., 2008; Swarnkar and Katewa, 2008; Sahu et al., 2010). Details of ethnobotanical values of different species of *Dioscorea* are listed in Table 1. Besides the traditional therapeutic values, many researchers reported other pharmacological/ common uses of different *Dioscorea* species.

## Food values of *Dioscorea* sp.

Since the adoption of the convention on biodiversity in 1992, there has been a general agreement on the importance of biodiversity, especially the diversity of wild and cultivated plants, to fill the need of the world population for food (FAO, 1998). In developing countries like India, people do not get enough food to meet their daily requirement, and most often the diet is deficient in one or more micronutrients (FAO, 1994). India faced a series of famines and major food shortages before 1940s. National food grain production was merely 50.82 million tons during 1950–1951, but has risen to 264.38 million tons in 2012–2013 (FAO, 1994). Edible roots and tubers not only enrich the diet due to the presence of starch and energy supplemented metabolites in them, but also possess medicinal properties due to the presence of diverse secondary metabolites. The tuber crop under study here, *Dioscorea*, is superior to many others as an important medico-food used by about 300 million people throughout the world (Arnau et al., 2010). In fact, they are one of the principal sources of energy food for many people in the Tropics (Nayaboga et al., 2014). As per source of dietary nutrients, *Dioscorea* species rank as the world's fourth most important root and tuber crops after potatoes, cassava, and sweet potatoes (Lev and Shriver, 1998). Many of the tubers of *Dioscorea* are bitter in taste, and local people use traditional skills to remove bitterness. Aboriginals also use their tubers as snacks, and in roasted, powdered, and other forms (Kumar et al., 2012; Misra et al., 2013). The same processes are also followed in various parts of India, including the Himalayan regions and North-Eastern part of India (Sheikh et al., 2013), Orissa (Sinha and Lakra, 2005; Kumar and Satpathy, 2011; Kumar et al., 2012; Misra et al., 2013), Tamil Nadu (Rajyalakshmi and Geervani, 1994; Shajeela et al., 2011), and among Palliyar and Kanikkar tribes (Shanthakumari et al., 2008; Arinathan et al., 2009) living in South-Eastern slopes of Western Ghats (Padmaja et al., 2001). They are also used by the local of Kumaon and Garhwal hills of India (Pramila et al., 1991). The nutrient content of the Yam has been compared with several other crops (Table 3; Wanasundera and Ravindran, 1994).

**Table 3:** **Nutrient content of Yam in comparison with other crops**.

**Crops**							
**Staple**	**Maize / Corn**	**Rice**	**Wheat**	**Potato**	**Cassava**	**Soybean (Green)**	**Yam**
**COMPONENT (PER 100G PORTION)**							
Water (g)	10	12	13	79	60	68	70
Protein (g)	9.4	7.1	12.6	2.0	1.4	13.0	1.5
Fat (g)	4.74	0.66	1.54	0.09	0.28	6.8	0.17
Carbohydrates (g)	74	80	71	17	38	11	28
Fiber (g)	7.3	1.3	12.2	2.2	1.8	4.2	4.1
Sugar (g)	0.64	0.12	0.41	0.78	1.7	0	0.5

Out of about 600 species, only 10 species of *Dioscorea* are cultivated throughout the world. In India, about 26 *Dioscorea* species are reported, and of them 13 are reported in SBR, Odisha (Kumar et al., 2012). Out of these 13 species, only one, *D. alata* L., is cultivated in this region, and the remaining species mostly grow wild in this zone. Tubers of 12 wild *Dioscorea* species available in Odisha have sound nutritional values, but have low palatability due to their bitterness (Kumar et al., 2012). *Dioscorea* the starchy edible tuber is of ample economic and nutritional importance in the Tropical and Sub-tropical regions of the world (Sharma and Bastakoti, 2009). In fact, the tubers are rich sources of food and energy (Table 3). The world's estimated yam production in 2010 was 48.7 million tons (Abasi et al., 2013). They have been reported to be good sources of essential dietary nutrients (Wanasundera and Ravindran, 1994).

## Bitterness and *Dioscorea* sp.

In spite of their nutritional importance, they possess some anti-nutritional factors and secondary metabolites, which make them bitter in taste and reduce the palatability. The major toxic content is dioscorine, an alkaloid present in most of the species of *Dioscorea* (Lu et al., 2012). Dioscorine triggers fatal paralysis of the nervous system (Reddy, 2015). Similarly, histamine was reported to be the principal allergen present, causing mild inflammation and itching (Shim and Oh, 2008). Some other compounds found in *Dioscorea* are furanoid-norditerpene, saponin, oxalate, tannin, and phytic acid. The primary bitter components present in the tubers of *D. bulbifera* L. have been identified by several researchers as the “furanoid norditerpenes” (diosbulbins A and B) (Martin, 1974; Webster et al., 1984; Bhandari and Kawabata, 2005). Therefore, various techniques are implemented to reduce or to eliminate the bitterness by the people. Most common techniques used to reduce the bitterness includes boiling/steaming and/or baking over coals after either cleaning (bulbils) or cleaning and peeling (tubers) (Bhandari and Kawabata, 2005). Martin (1974) documented that the tubers of several toxic varieties of *D. bulbifera* L. are made palatable by the above said techniques and are used as a source of food during the extreme conditions such as drought and or famine. In some places, the detoxification is done with lime or sand and then slow-roasting or repeated boiling with wood ashes followed by steeping sliced pieces in running water (Martin, 1974; Webster et al., 1984).

## Pharmacological potential of *Dioscorea* sp.

Studies reveal that the tubers of *Dioscorea* species possess high amounts of polyphenolic compounds (Liu et al., 2011). Several researchers have reported the pharmacological importance of *Dioscorea* species (Kumar and Jena, 2014). *Dioscorea* species have been reported to have anti-oxidative, anti-fungal, anti-mutagenic, hypoglycaemic, and immunomodulary effects (Son et al., 2007). They are also used as important ingredients of dietary supplements and in cosmetics and pharmaceutical industries (Black et al., 2007). Tubers and other parts of *Dioscorea* possess different types of phenolic compounds, which might be responsible for the antimicrobial activities (Sara et al., 2010). Aderiye et al. (1996) reported the antifungal activity of *D. alata* L. peel extracts.

In the year 2003, Seetharam et al. (2003) documented the antimicrobial activity of D. bulbifera L. (bulbil) against Klebsiella pneumoniae, Escherichia coli, Proteus vulgaris, Staphylococcus aureus, Aspergillus niger, Aspergillus flavus, Aspergillus fumigatus, and Rhizopus nigricans. Many authors have reported the antimicrobial potential of different species of Dioscorea sp. in different studies such as antibacterial activities of D. zingiberensis C.H. Wright by Xu et al. (2008); antimicrobial activity of D. hamiltonii Hook.f. tubers with *Azadirachta indica* stem by Kaladhar et al. (2010); the antibacterial activity of *D. pentaphylla* L. against *S. aureus, P. aeruginosa*, and *K. pneumoniae*, and anti-fungal activity against *Trichophyton rubrum, Microsporum gypseum, Trichophyton tonsurans, Microsporum audouini*, and *Candida albicans* found in the mid-Western Ghats by Prakash and Hosetti (2010). The antibacterial activity of *D. villosa* L. tubers against *S. dysenteriae, E. coli, V. cholerae, K. pneumoniae, P. aeruginosa*, and *S. aureus* was also reported (Roy et al., 2012).

The silver nano-particles synthesized from *Dioscorea* tuber extracts were found to possess potent synergistic antibacterial activity with combination of antibiotics (Ghosh et al., 2012). The folkloric uses of the studied plants and provided evidence that tuber extracts of *D. dumetorum* (Kunth) Pax and *D. hirtiflora* Benth. might indeed be potential sources of natural antioxidant and antimicrobial agents in Nigeria as reported by Sonibare and Abegunde (2012). Chandra et al. (2013) documented the antimicrobial activity of all extracts of *D. deltoidea* Wall. ex Griseb. against ten (gram negative and gram positive) bacteria and three fungal strains. Begum and Anbazhakan (2013) described the antimicrobial activity of tuber mucilage extracted from *D. esculenta* (Lour.) Burkill by *in vitro*-well diffusion assay against *E. coli, K. pneumoniae, P. aeruginosa, S. aureus*, and *Streptococcus pyogenes*. The extracts showed significant antibacterial activity against *E. coli, P. aeruginosa*, and *S. aureus*, while no activity was observed by the extracts against *K. pneumoniae* and *S. pyogenes*.

Many studies have also shown the anti-oxidant potentials of these tuber crops. Araghiniknam et al. (1996) observed the antioxidant activity of *Dioscorea* species. Hou et al. (2001) documented the antioxidant activity of dioscorin of yam (*D. batatas* Decne.) tubers. Hou et al. (2002) also reported the antioxidant activity of yam (*D. batatas* Decne.) tuber mucilage. In the year 2004, Dong et al. (2004) testified steroidal saponins from *D. panthaica* Prain & Burkill and their cytotoxic activity from an ethanol extract of the rhizomes of *D. panthaica* Prain & Burkill and in the same year, Yu et al. (2004) have also stated the anticancer effects of various fractions extracted from *D. bulbifera* L. on mice bearing HepA. Further, Chang et al. (2004) have described that Chinese yam (*D. alata* L.) had antioxidative effects in hyperhomocysteinemia rats. Similarly, Liu et al. (2006) documented the antioxidant activities of selected *Dioscorea* species using DPPH radical, hydroxyl radical scavenging activity assay, and anti-lipid peroxidation test.

In the year 2002, Gao et al. (2002) observed the anti-tumor-promoting effect with ethanol extracts of the tubers of *D. bulbifera* L. using the neoplastic transformation assay of mouse epidermal JB6 cell lines. Later, Oh and Lim (2008) testified the antioxidant activity of glycoproteins isolated from *D. batatas* Decne. Theersin and Baker (2009) identified various phenolic compounds in *D. hispida* Dennst. and analyzed their antioxidant potential while Sonibare and Abegunde (2012) reported the antioxidant activity and the bioactive metabolites of *Dioscorea* species of Nigeria. The antitumor and antioxidant potential of *D. bulbifera* L. and *D. esculenta* (Lour.) Burkill have also been reported (Murugan and Mohan, 2012; Wang et al., 2012). In 2016, Zhang et al. (2016) documented the antioxidant and antimutagenic activity of the mucilage polysaccharide of *D. oppositifolia* L. while in the same year, Liu et al. (2016) showed the antioxidant and antitumor activities of the extracts from the flesh and peel of Chinese Yam (*D. opposite* Thunb.).

## Bioactive compounds present in *Dioscorea* sp.

**Figure 3:**
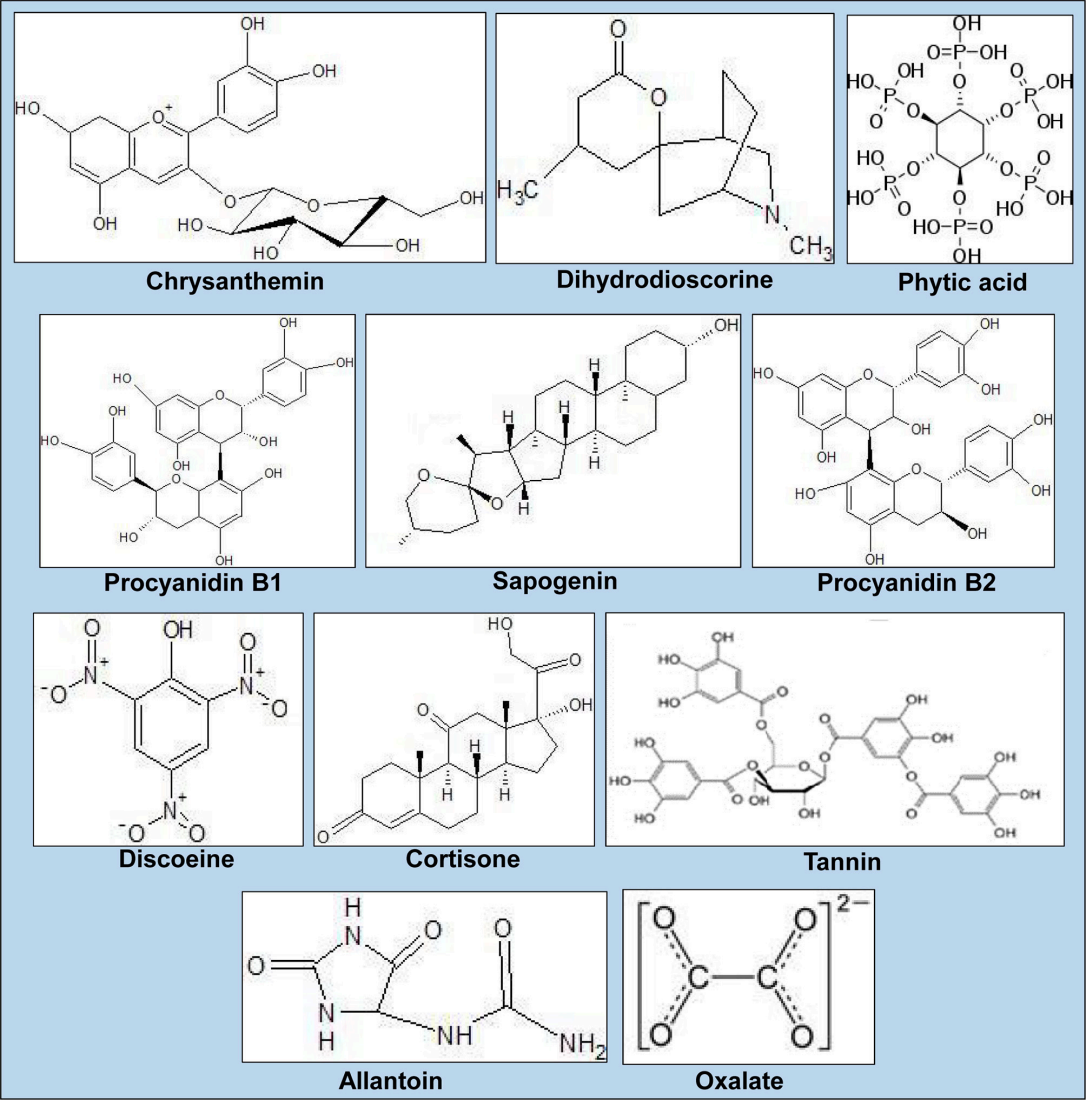
**Chemical structure of selected bioactive compounds from ***Dioscorea*** species with medicinal potential**.

**Figure 4:**
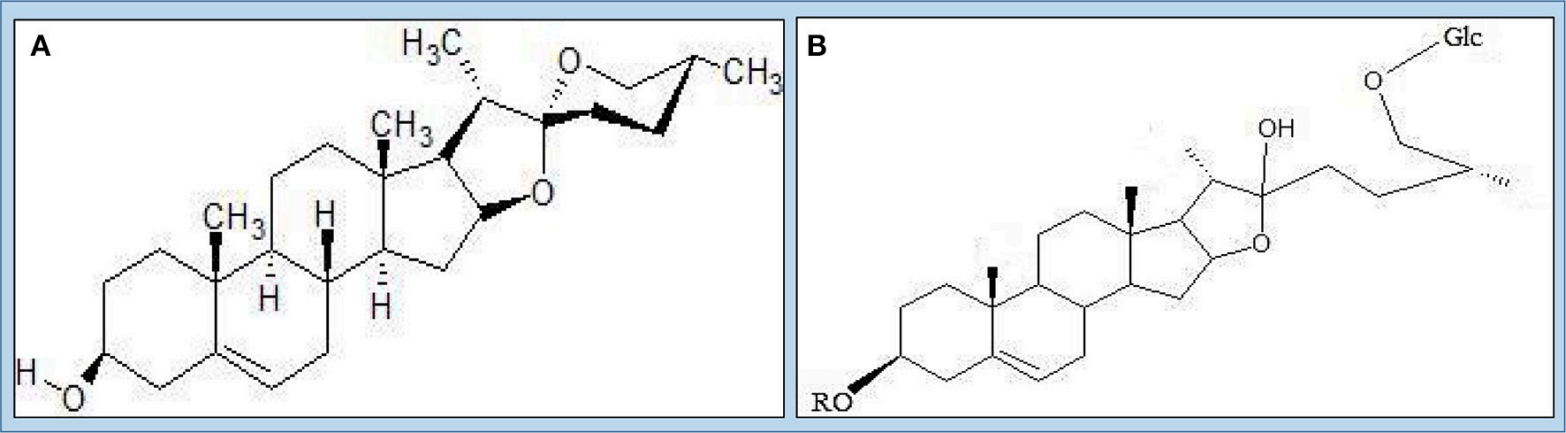
**The structure of Diosgenin, (A)** Protodioscin; **(B)** Steroid saponins.

The tuber and other parts of *Dioscorea* species possess different types of bioactive compounds (Table 4, Figure 3) so that its parts are used against different diseases. These bioactive compounds also reflect the indigenous therapeutic values among the many races of aboriginals of the world (Nayaboga et al., 2014). All these factors make them sound pharmacological agents and good sources for isolation and formulation of new compounds that can fight against different types of diseases. The most important component reported by Martin (1969) was diosgenin, a sapogenin used in the synthesis of steroidal drugs. Diosgenin is the primary active ingredient in *Dioscorea* species. It is structurally similar to cholesterol. After oral administration, it is metabolized in the liver and eliminated via the bile (Caven and Dvornik, 1979). Estrogenic and anti-inflammatory effects of diosgenin have been hypothesized due to its structural similarity to estrogen precursors. Asha and Nair (2005) reported that *D. deltoidea* Wall. ex Griseb. is the major species exploited in India for diosgenin (Figure 4) production from rhizomes.

**Table 4:** **Common bioactive compounds present in ***Dioscorea*** species of SBR, India**.

**Compounds**	**Uses**	**Species**	**Source(s)**
Diosgenin	Synthesis of steroidal drugs	*Dioscorea deltoidea* Wall. ex Griseb.	Asha and Nair, 2005
Sapogenin	Anti-inflammatory effect	*Dioscorea* spp.	Martin, 1969
Saponin	Skin infections	*Dioscorea* spp.	Nayaboga et al., 2014
Cyanidin	Exhibit trypsin inhibitors	*Dioscorea* spp.	Hou et al., 2000
Flavonoids	Skin infections	*Dioscorea belophylla* (Prain) Voigt ex Haines	Poornima and Ravishankar, 2007
Allantoin	Detoxification of ammonia	*Dioscorea* spp.	Fujihara and Yamaguchi, 1978
Dioscorine	Birth control	*Dioscorea bulbifera* L.	Adetoun and Ikotun, 1989
Ohenolic compounds	Skin infections	*Dioscorea pentaphylla* L.	Kumar and Jena, 2014

Asha and Nair (2005) also reported the maximum diosgenin yield in *D. pubera* Blume followed by *D. spicata, D. hispida* Dennst. and *D. hamiltonii* Hook.f. Ozo et al. (1984) reported phenolic compounds Cyanidin-3-glucoside and the procyanidin dimmers B-1 and B-3 from *D. alata* L. Hou et al. (2000) reported that dioscorins present in *Dioscorea* species have potential to exhibit carbonic anahydrase and trypsin inhibition activities. Okunlola and Odeku (2009) reported the compression characteristics and tableting properties of starches of *D. dumetorum* (Kunth) Pax, *D. alata* L., *D. oppositifolia* L., and *D. rotundata* Poir. Avula et al. (2012) reported 20 different types of steroidal saponins from *Dioscorea* species using UHPLC-QTOF-MS. Franklin and Cabanillas (1966) reported the production of sapogenin in different *Dioscorea* species. Martin and Cabanillas (1963) reported a precursor of cortisone and related steroidal drugs from *Dioscorea* species.

Poornima and Ravishankar (2007) reported the bioactive compounds in *D. belophylla* (Prain) Voigt ex Haines as saponins, alkaloids, flavonoids, tannin, and phenols. Yoon et al. (2008) reported a bioactive compound, Allantoin, in *Dioscorea* rhizomes. Bhandari and Kawabata (2005) identified bitter components in *Dioscorea* as Furanoid norditerpenes cyanogens. Martin (1974) reported the yellow pigments of *D. bulbifera* L. and that the yellow color is due to the presence of saponifiable of xanthophylls such as Lutein. The phytochemicals studies also revealed that purine derivatives, saponin, starches, and mucilage are the main constituents in *Dioscorea* tubers, along with allantoin, a purine derivative (Fu et al., 2006). Allantoin is present as a nitrogen storage form in plants or as a product of detoxification process of ammonia in plant tissues (Fujihara and Yamaguchi, 1978). It has been also demonstrated that *Dioscorea* species contain higher level of allantoin than any other plants (Fu et al., 2006). Therefore, allantoin could be a good standard substance for the quality control of *Dioscorea* tubers because of their pharmacological importance and abundance in *Diosorea* species. There have been many reports to determine allantoin in biofluid with HPLC, LC-MS/MS, GC-MS (Berthemy et al., 1999; Czauderna and Kowaleczyk, 2000), and a capillary electrophoresis method for measuring allantoin content in *Dioscorea* tubers. *D. bulbifera* L. has more allantoin content than other species, which inhibits the α-amylase and α-glucosidase activity responsible for its anti-diabetic action. It has anti-oxidant, anti-hyperglycemic, and anti-dyslipidemic activities. Methanol extract of *D. oppositifolia* L. has anti-ulcer activity. In 1989, Adetoun and his co-workers reported that bulbils and tubers of *D. bulbifera* L. contained an alkaloid, dihydrodioscorine. When crystallized in its hydrochloride form and incorporated into potato dextrose agar at a final concentration of 0.1%, it was found to slow down the rate of growth of five plant pathogenic fungi: *S*. *rolfsii, C. lunata, F. moniliforme, M. phaseolina*, and *B. theobromae*.

## Conclusion and future research needs

Little research attention, minimal commercialization, and deficient policy frameworks are the major obstacles to harnessing the actual biodiversity potential of SBR. Of the phytodiversity of SBR, tubers and root crops are important so far as their uses are concerned. The wild tubers also act as a “safety net” for local people during their critical time of drought and famine. Among the tuberous wild edible medicines, *Dioscorea* species are quite common. They are a prime staple food substitute for the majority of rural and local people of SBR. The plant parts are quite useful in treatment of different types of diseases and disorders due the presence of a numbers of bioactive compounds. The ethnomedicinal potential of various plant species under this genus need to be validated and detailed investigations on the composition and pharmacological significance of the medicinal plants under this genus along with the standardization of the formulations used should be undertaken extensively.

The most important identified compound from *Dioscorea* species is diosgenin, it is presently used in the synthesis of steroidal drugs, however other potential uses of this compounds and related compounds as estrogenic, anti-inflammatory and anticancer potential need to be studied extensively. Similarly authentication of all the secondary metabolites (alkaloids, saponin, flavonoids, tannins and phenols) from this genus should be performed carefully by advanced analytical techniques to validate its quality and for conforming its biological potentials. Further studies are also required to address various issues regarding the composition of the extracts used, explicability of the preclinical experiments and lack of conversion of the preclinical results to clinical effectiveness. Attempt should also be made to conduct serious human trails and to determine the mechanism of action, bioavailability, pharmacokinetics and the physiological pathways for various types of bioactive compounds for their potential applications in drug discovery and for curing various life threatening diseases. Studies should also be carried out to utilize the bioactive compounds present in these tubers for formulation of new drugs to fight against pathogenic multidrug resistant microorganisms and antimicrobial resistance. Research on these crops will open up new vistas in the study of biodiversity management for sustainable development, germplasm conservation, pharmacology and many other maiden fields of research in plant science and pharmaceutics.

## Author contributions

Draft preparation: SK, JP, GD, and HS; Paper writing: SK, GD, JP; Editing: JP, HS. All authors read and approved the final manuscript.

### Conflict of interest statement

The authors declare that the research was conducted in the absence of any commercial or financial relationships that could be construed as a potential conflict of interest.
